# High Prevalence and Genetic Diversity of HCV among HIV-1 Infected People from Various High-Risk Groups in China

**DOI:** 10.1371/journal.pone.0010631

**Published:** 2010-05-27

**Authors:** Hong Shang, Ping Zhong, Jing Liu, Xiaoxu Han, Di Dai, Min Zhang, Ke Zhao, Rongzhen Xu, Xiao-Fang Yu

**Affiliations:** 1 Key Laboratory of AIDS Immunology of Ministry of Health, No 1 Hospital of China Medical University, Shenyang, Liaoning, China; 2 Shanghai Municipal Center for Disease Control and Prevention, Shanghai, China; 3 Center for Virus and AIDS Research, Jilin University First Hospital, Changchun, Jilin, China; 4 W. Harry Feinstone Department of Molecular Microbiology & Immunology, Johns Hopkins University Bloomberg School of Public Health, Baltimore, Maryland, United States of America; 5 Second Affiliated Hospital, School of Medicine, Zhejiang University, Hangzhou, Zhejiang, China; Tsinghua University, China

## Abstract

**Background:**

Co-infection with HIV-1 and HCV is a significant global public health problem and a major consideration for anti-HIV-1 treatment. HCV infection among HIV-1 positive people who are eligible for the newly launched nationwide anti-HIV-1 treatment program in China has not been well characterized.

**Methodology:**

A nationwide survey of HIV-1 positive injection drug uses (IDU), former paid blood donors (FBD), and sexually transmitted cases from multiple provinces including the four most affected provinces in China was conducted. HCV prevalence and genetic diversity were determined. We found that IDU and FBD have extremely high rates of HCV infection (97% and 93%, respectively). Surprisingly, people who acquired HIV-1 through sexual contact also had a higher rate of HCV infection (20%) than the general population. HIV-1 subtype and HCV genotypes were amazingly similar among FBD from multiple provinces stretching from Central to Northeast China. However, although patterns of overland trafficking of heroin and distinct HIV-1 subtypes could be detected among IDU, HCV genotypes of IDU were more diverse and exhibited significant regional differences.

**Conclusion:**

Emerging HIV-1 and HCV co-infection and possible sexual transmission of HCV in China require urgent prevention measures and should be taken into consideration in the nationwide antiretroviral treatment program.

## Introduction

Hepatitis C virus (HCV) is a single-stranded RNA flavivirus that was first recognized as the cause of non-A-non-B hepatitis [1], [2]. The HCV epidemic now affects over 200 million people worldwide. Co-infection with HCV and HIV-1 is also a significant global public health problem and has been shown to increase HCV viral loads and promote the development of cirrhosis and progression to end-stage liver disease [3]–[6]. Whether co-infection with HIV-1 and HCV results in a faster progression to AIDS remains controversial [7], [8]. More importantly, co-infection with these two viruses will continue to cause increased morbidity and mortality in many developing countries, where treatment for either virus is still largely unavailable [9], [10].

In China, the HIV-1 epidemic has been carefully followed among injection drug users (IDUs) since its emergence in Yunnan Province in the late 1980s [11], [12]. More recently, emerging HIV-1 epidemics among IDUs in other parts of China have been linked to heroin trafficking routes [13]–[18]. HIV-1 infection was also a serious problem among former paid blood donors in China during 1990s and is now spreading among people who are sex partners of high-risk populations [19], [20]. Relatively less is known about the spread of HCV infection and HCV/HIV-1 co-infection in China [21]–[24]. Few population-based studies have been performed in China to systematically examine co-infection with HIV-1 and HCV in IDUs, former paid blood donors (FBD), or those who have acquired HIV-1 through sexual contact (SC). The distribution of HCV genotypes among various risk groups has not been systematically evaluated. It is also not clear how widespread the HCV epidemic is in China and whether there is any spreading pattern of HCV infection.

The aim of the present study was to investigate the problem of HCV infection among HIV-1-infected people from various risk groups in China and to determine the HCV genotype distribution in these individuals. High rates of HCV infection were found among HIV-1-infected people from all high risk groups including IDUs, PBDs, and sexually transmitted cases. Distinct HIV-1 and HCV genotypes were identified according to risk groups and regional differences in the distribution of HCV genotypes among IDUs were observed. Our data have important implications for HIV-1 and HCV transmission and treatment in China.

## Results

### High prevalence of HCV infection among HIV-1-positive subjects in high-risk groups in China

**Figure 1:**
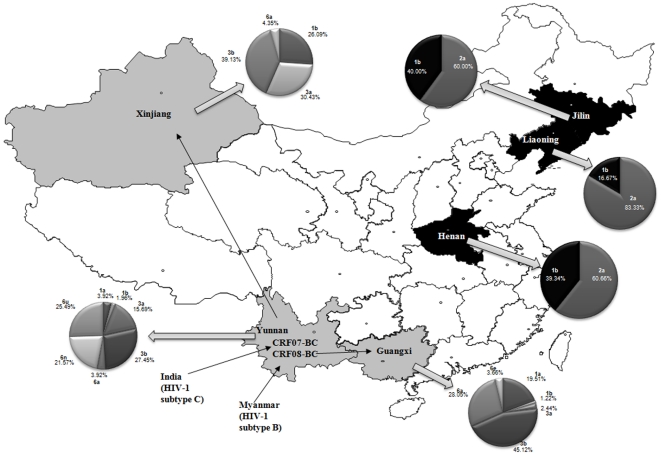
Map of HIV-1 and HCV distribution in China. IDUs from Yunnan, Guangxi, and Xinjiang provinces were studied because injection drug use and HIV-1 infection are most problematic in these provinces in China [20]. Spreading of CRF07 and CRF08 among IDUs has been detected in Yunnan, Guangxi, and Xinjiang provinces in China along the drug trafficking routes (arrows). However, HCV genotypes among IDUs from these provinces are distinct (pie charts). HCV genotyping among FBD in Henan, Jilin, and Liaoning provinces showed another difference from IDU.

To systematically evaluate the problem of HIV-1/HCV co-infection, we analyzed samples from a total of 178 HIV-1-infected IDUs from Yunnan, Xinjiang, and Guangxi provinces, 128 FBD from Henan, Jilin, and Liaoning provinces, and 159 SC from the same six provinces (Fig. 1). IDUs from Yunnan, Guangxi, and Xinjiang provinces were studied because injection drug use and HIV-1 infection are most problematic in these provinces in China [20]. Although HIV-1 infection among FBD is known to be a major problem in Henan province, the HIV-1 infection that has also been detected among FBD in the Northeast provinces Jilin and Liaoning has not been well characterized to date (Fig. 1).

We found that the prevalence of HCV in HIV-1-infected subjects was 96.6% in IDUs and 92.9% in FBD (Table 1). There was no significant difference in HCV prevalence among IDUs from different provinces (Table 2). The prevalence of HCV infection among HIV-1 positive FBD from Henan was somewhat higher than that from Liaonin, or Jilin provinces (Table 2). The extremely high prevalence of HCV among HIV-1-positive IDUs and FBD is consistent with the common transmission mechanism of both viruses. Remarkably, we also detected high rate of HCV infection (20.1%) among people who acquired HIV-1 infection through sexual contacts i.e. SC subjects (Table 1). Although the HCV prevalence among HIV-1-positive SC subjects was lower than that in IDUs and FBD, it was still significantly higher than the HCV prevalence reported for the general population. For example, the HCV prevalence in more than 160,000 voluntary blood donors from large cities in China such as Beijing, Chongqing, and Jinan has been reported to be <1% [25]–[27]. We have also observed low HCV prevalence rates among 10,078 outpatients and inpatients in a Liaoning provincial hospital (Table 1). Furthermore, among 30,296 subjects who underwent routing health examination, only 166 (0.55%) were found to be HCV positive. Thus, HIV-1-infected people apparently have high rates of HCV co-infection, regardless of the risk factors for HIV-1 acquisition.

**Table 1:** HCV prevalence among HIV-1 positive subjects from various risk groups in China.

HIV-1+	HCV+	Mean Age	Sex male/femal
Injection drug users (IDU)	96.6% (172/178)	28.9±9.7	162/10
Former paid plasma donor (FPD)	92.9% (119/128)	42.6±9.4	73/46
People who acquired HIV-1 through sexual contact (SC)	20.1% (32/159)	37.9±9.0	16/16
Hospital outpatients	1.55% (87/5610)		
Hospital inpatientsRouting health examination	1.23% (55/4468)0.55% (166/30,294)		

General outpatients and inpatients were also evaluated as controls.

**Table 2:** HCV prevalence among HIV-1 positive subjects from different provinces in China.

Risk factors	Location	Risk Locationfactors			
FBD			IDU		
	Henan	96.6% (84/87)		Guangxi	95.0% (96/101)
	Liaonin	86.7% (13/15)		Xinjiang	95. 8% (23/24)
	Jilin	84.6%(22/26)		Yunnan	100%(53/53)

### Distinct HIV-1 subtypes among people from different risk groups

Overland drug trafficking has been linked to the emergence of novel recombinant strains of HIV-1 and the spread of HIV-1 infection among drug users in China [13]–[18]. CRF-07 and CRF-08 are subtype B and C recombinant viruses (CRF-BC) that have their origin in Yunnan province [13]–[18]. CRF07 spread along the ancient silk road to Xinjiang from Yunnan among IDUs, and CRF08 spread eastward from Yunnan to Guangxi province [13]–[18]. HIV-1 subtypes were determined for 127 (IDUs) from Yunnan, Guangxi, and Xinjiang provinces. Consistent with previous reports [13]–[18], CRF-BC recombinant HIV-1 strains were the predominant HIV-1 strains in IDUs in areas along these shared drug trafficking routes (Table 3). On the other hand, HIV-1 subtype B' was the predominant HIV-1 subtype among FBD in Henan, consistent with previous observations [22]. For the first time, we have observed that HIV-1 subtype B' is also the predominant HIV-1 subtype among FBD in the Northeast provinces of Liaonin and Jilin (Table 3). Since Henan and Jilin provinces are far away (>1,000 km) from each other, our data suggest that certain paid former blood donors may travel long distance from one place to another, causing the spread of HIV-1 infection in the same risk group. Overall, distinct HIV-1 subtypes were confined within the IDU and FBD populations from geographically separated provinces in China.

**Table 3:** Distribution of HIV-1 subtypes in different provinces from China.

Risk factors	Location	HIV-1 subtypes					
		B'	B	CRF01-AE	CRF-BC	CRF02-AG	C
FBD							
	Henan	84	3	0	0	0	0
	Liaonin	15	0	0	0	0	0
	Jilin	26	0	0	0	0	0
IDU							
	Guangxi	0	0	2	48	0	0
	Xinjiang	0	0	0	24	0	0
	Yunnan	0	0	1	52	0	0

### HCV genotyping

**Figure 2:**
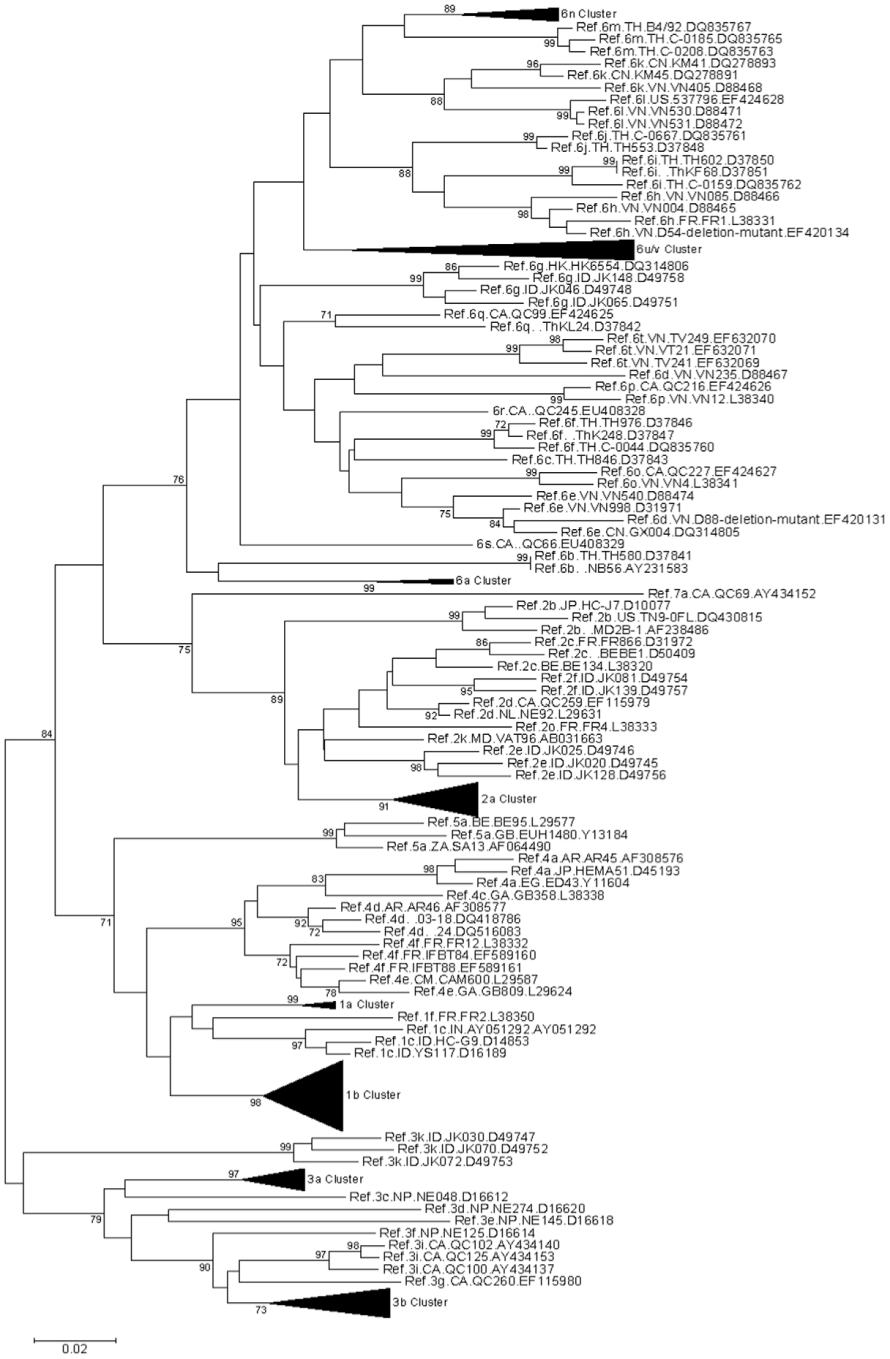
Neighbor-joining tree demonstrating that Hepatitis C virus sequences from China belong to 1a, 1b, 2a, 3a, 3b, and 6 genotypes. Sequences analyzed here contain 306 nucleotides, corresponding to 350–655 of H77 genome. The tree was drawn with MEGA 4 (Build #4028)[36], using Kimura 2-parameter as the model. The references were obtained from the Los Alamos National Laboratory HCV Databases (http://hcv.lanl.gov/content/index). Bootstrap values >70% are indicated at the nodes of the corresponding branches.

Molecular epidemiology of HIV-1 infection has been monitored since the beginning of the HIV-1 epidemic in China. This analysis has led to the identification of emerging transmission of HIV-1 from IDUs in Yunnan to those in Xinjiang and Guangxi [13]–[18]. To determine whether HCV genotyping could also be used to monitor the pattern of HCV transmission and drug trafficking, HCV genotypes were determined and compared for IDUs from Yunnan, Xinjiang, and Guangxi provinces. Phylogenetic analysis of HCV sequences from China indicated the existence of HCV 1a, 1b, 2a, 3a, 3b, and various 6 genotypes (Fig. 2 and supplemental Figures S1, S2 and S3).

Samples from Yunnan IDUs were classified into genotypes 1a, 1b, 3a, 3b, and various 6 genotypes (Table 4). Genotype 6 was the major HCV genotype (>50%) in IDUs in Yunnan. However, genotype 6 was almost absent (in only 1 of 23 individuals) from IDUs in Xinjiang. On the other hand, genotype 3 HCV accounted for >70% of all IDUs in Xinjiang, as compared to 40% of IDUs in Yunnan. Furthermore, although genotype 6 was detected among IDUs in both Yunnan and Guangxi, IDUs in Guangxi were mostly genotype 6a, while IDUs in Yunnan were mostly genotype 6n and a new genotype 6u (Table 4).

**Table 4:** Distribution of HCV genotypes in different provinces from China.

Risk factors	Location	HCV genotypes								
		1a	1b	2a	3a	3b	6a	6e	6n	6u/v
FBD	Henan	0	37	24	0	0	0			
	Liaoning	0	5	1	0	0	0			
	Jilin	0	8	4	0	0	0			
										
IDU	Yunnan	2	1	0	8	14	2		11	13
	Xinjiang	0	6	0	7	9	1			
	Guangxi	11	1	0	1	24	8	2		
										
Sex	Liaoning	0	1	0	0	0	0			
	Henan	0	5	2	0	1	0			
	Xinjiang	0	3	0	2	1	0			
	Yunnan	1	0	0	0	1	0			1

HCV genotypes in FBD from central and northeast China were more homogenous than were those in IDUs. Only genotypes 1b and 2a were detected, and genotype 1b was the predominant form in FBD from all three provinces (Table 4). Surprisingly, although diverse HCV genotypes were detected among IDUs in China, genotype 2a HCV was completely absent from all the IDUs examined.

**Figure 3:**
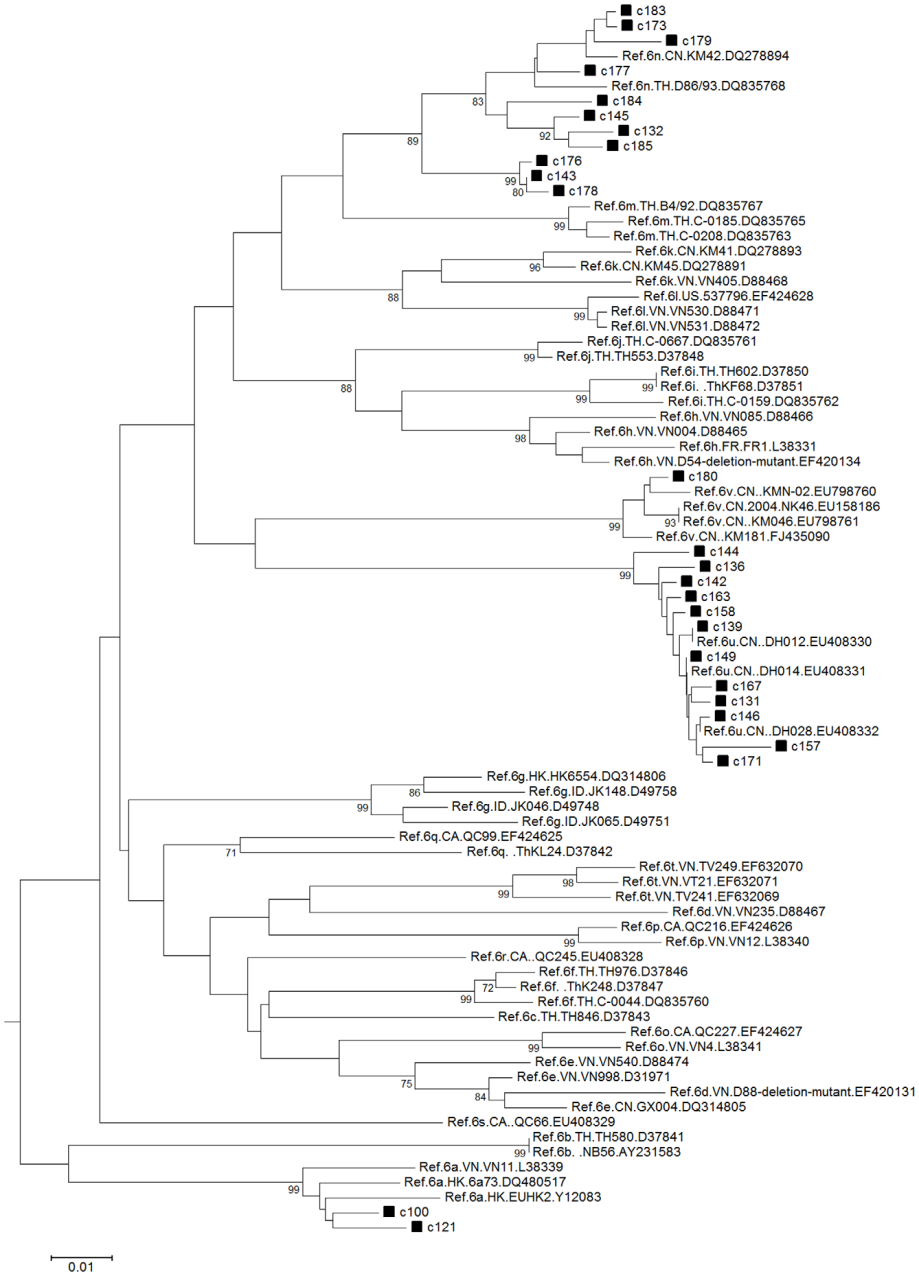
HCV genotype 6 phylogenetic tree of injection drug users from Yunnan province, China. Sequences analyzed here contain 306 nucleotides, corresponding to 350–655 of H77 genome. The tree was drawn with MEGA 4 (Build #4028), using Kimura 2-parameter as the model. The references were obtained from the Los Alamos National Laboratory HCV Databases (http://hcv.lanl.gov/content/index). Bootstrap values >70% are indicated at the nodes of the corresponding branches. Symbol ▪ indicates the sequence is from an IDU sample.

The detection of diverse HCV genotype 6 sequences in Yunnan was interesting (Table 4). Thus the phylogenetic analysis of these diverse HCV genotype 6 sequences was performed using well characterized genotype 6 reference sequences (Fig. 3). A cluster of genotype 6 sequences grouped with the recently identified genotype 6u sequences [28]–[30]. One sequence grouped with the recently identified genotype 6v sequences [31]. Furthermore, although some sequences clustered with genotype 6n, several sequences, such as c143, c176, and c178, fell between 6n and 6m. Thus, the diversity of genotype 6 HCV sequences from Yunnan was quite remarkable.

## Discussion

Injection drug use in China was the driving force for the original HIV-1 epidemic and continues to be a major source of HIV-1 infection [19], [20]. Yunnan, Xinjiang, and Guangxi provinces carry the majority of the HIV-1 cases among IDUs in China. During the 1990s, HIV-1 infection in FBD in central China became another major source of HIV-1 infection in China [19], [20]. Although many cases have been reported from Henan Province, FBD-related HIV-1 infections have also affected other provinces, such as Liaoning and Jilin in the northeast region of China. In recent years, sexual transmission of HIV-1 in China has also increased significantly [19], [20] and now represents a major threat to the general population.

The current study indicates that HCV infection is a major problem for HIV-1-infected individuals, regardless of the route of transmission: injection drug use, paid blood donation, or sexual contact. The high rate of HCV and HIV-1 co-infection is a major public health concern. HCV is now a major opportunistic infection for those people who are infected with HIV-1. Previous studies have shown that HIV-1/HCV co-infections increase the HCV viral load and progression to end-stage liver disease (ESLD), hepatocarcinoma, and hepatic-related death. HCV infection has also been reported to result in a higher risk of AIDS-defining illnesses, AIDS-related death, and hospitalization in HCV/HIV-1 co-infected patients.

It is estimated that more than 30,000 HIV-1-infected patients have been enrolled in the nationwide antiretroviral treatment program in China [19]. HCV positive subjects in China have low viral clearance rates [32]. The high rate of co-infection with HCV among HIV-1-infected patients represents a major challenge for the treatment of HIV-1-infected people in China [10], [33]. HCV-related liver disease has emerged as a leading cause of death in people who undergo highly active antiretroviral treatment [10], [33]. Thus, HIV-1 mono-therapy may have limited benefit for HCV/HIV-1 co-infected patients. Furthermore, the efficacy and liver toxicity of anti-HIV-1 drugs in these co-infected patients have to be considered and carefully monitored during HIV-1 treatment [34], [35].

Sexual transmission of HCV has been documented [10], [33]. However, the frequency and extent of this sexual transmission remain controversial. Our study has revealed a significantly high prevalence of HCV infection among HIV-1-positive subjects who acquired HIV-1 through sexual contact. The HCV prevalence (20%) among HIV-1-positive SC subjects was clearly higher than that in volunteer blood donors (<1%) from various provinces across China. We have also found that both inpatients and outpatients in a Liaoning hospital have much lower HCV prevalence than do SC. Strategies to reduce the transmission of HCV among spouses and sex partners of HCV-positive people are urgently needed.

Overland heroin trafficking routes have been associated with dual epidemics of injection drug use and HIV-1 infection in China [13]–[18]. The molecular epidemiology of HIV-1 subtypes among IDUs from several provinces, including Yunnan, Guangxi, and Xinjiang, has indicated that the CRF07 and CRF08 BC recombinant viruses have spread along distinct drug trafficking routes. In IDUs, HCV is transmitted through risk behaviors similar to those that transmit HIV-1. However, the HCV genotypes in IDUs from different provinces did not match the patterns of HIV-1 transmission and drug trafficking. For example, the most common HCV genotype detected among IDUs in Yunnan was genotype 6 (6n and 6u). However, genotype 6 HCV was almost absent from IDUs from Xinjiang, where genotype 3 (3a and 3b) accounted for about 70% of all HCV infections. In Guangxi, genotype 6 was also less prevalent among IDUs (Table 3). Furthermore, although genotype 6 was detected among IDUs in both Yunnan and Guangxi, IDUs in Guangxi were mostly genotype 6a, while IDUs in Yunnan were mostly genotypes 6n and 6u of HCV (Fig. 2). Thus, while molecular epidemiology of HIV-1 is useful for tracking the spreading pattern of HIV-1 infection and heroin trafficking routes in China, this approach may prove less helpful for HCV molecular genotyping. The high diversity of HCV genotypes that we observed in China may also have implications for future HCV treatment.

## Methods

### Study participants and serological testing

HIV-1 positive subjects from several major provinces in China were recruited between 2000 and 2008 by the Provincial Center for Disease Control. This study was approved by the institution's ethical committee. The study participants were interviewed by clinicians to determine their epidemiologic background, using questionnaires designed by the UNAIDS HIV-1 characterization network. Study subjects included 159 individuals with sexual risk, 128 former paid blood donors (FBD), and 178 injection drug users (IDUs). The presence of HIV-1 antibody was determined by enzyme-linked immunosorbent assay (ELISA) using the Vironostika HIV-1 Microelisa System (Organon Teknika). ELISA-positive samples were not considered HIV-1-positive until confirmation by the HIV-1/2 Western blot immune assay (Gene Lab, Singapore). Hepatitis C antibody was analyzed using the Ortho HCV Version 3.0 ELISA Test System (Ortho Diagnostic Systems, Raritan, NJ, USA).

### RNA purification, PCR amplification, sequencing, and phylogenetic analysis

HIV-1 RNA was extracted from plasma by a column purification method (QIAamp® Viral RNA Mini Kit, Qiagen, Hilden, Germany) and subjected to reverse transcription-nested PCR. The nucleotide sequence of the 2.6-kb *gag*-reverse transcriptase (RT) gene (nucleotide position relative to HXB2 genome: 790-3421) was determined by direct sequencing (ABI PRISM® 3130 Genetic Analyzer, Applied Biosystems, Foster City, CA, USA). The PCR primer-binding sites for the amplicon were based on published sequences of geographical variants and were as highly conserved as possible. Codon alignment of query sequences with various HIV-1 reference subtypes and CRFs from the HIV-1 database (http://HIV-web.lanl.gov/) was performed. Selected HIV-1 subtypes/CRFs of geographical importance were also included in the alignment. Phylogenetic and molecular evolutionary distances were estimated using the neighbor-joining method and the Kimura 2-parameter model with a transition-transversion ratio of 2.0. The reliability of the branching orders was tested by bootstrap analysis of 1,000 replicates. Bootscanning, similarity plots, and informative site analysis were performed using SimPlot v3.5[11] to define the recombination structure. All RNA extractions and PCR amplifications were carried out with appropriate negative controls to detect possible contamination during the procedure. To check for potential contamination, the sequences obtained were compared to all known sequences in the HIV-1 database by a BLAST search (http://HIV-web.lanl.gov/content/index) prior to analysis.

RNA for HCV genotyping was extracted from 100 µL of serum using the QIAamp Viral RNA kit (QIAGEN Inc, Valencia, CA, USA). Reverse transcription and nested PCR were performed using primers for conserved regions of Core as previously described [23]. After purification with the QIAquick PCR Purification kit (QIAGEN Inc, Valencia, CA), samples were sequenced using the inner forward primer on an automated sequencer (PRISM, version 2.1.1; ABI). Sequences were compiled using the BioEdit program, version 4.7 (T. Hall, North Carolina State University, Raleigh), and genotypes were assigned after alignment with known HCV genotypes as previously described [23].

### Ethics Approval

Written consents were obtained from all participants involved in the study. This activity was reviewed and approved by the institutional review board of the First Hospital of Chinese Medical University in Shenyang, China.

## Supporting Information

Figure S1 Neighbor-joining subtree demonstrating that Hepatitis C virus sequences from China belong to genotype 1. The tree was drawn with MEGA 4 (Build #4028), using Kimura 2-parameter as the model. The references were obtained from the Los Alamos National Laboratory HCV Databases (http://hcv.lanl.gov/content/index). Bootstrap values >70% are indicated at the nodes of the corresponding branches. Symbol • indicates the sequence is from a PBD sample, while ▪ is from IDU.
(0.82 MB JPG)

Figure S2 Neighbor-joining subtree demonstrating that Hepatitis C virus sequences from China belong to genotype 2. The tree was drawn with MEGA 4 (Build #4028), using Kimura 2-parameter as the model. The references were obtained from the Los Alamos National Laboratory HCV Databases (http://hcv.lanl.gov/content/index). Bootstrap values >70% are indicated at the nodes of the corresponding branches. Symbol • indicates the sequence is from a PBD sample, while ▪ is from IDU.
(0.75 MB JPG)

Figure S3 Neighbor-joining subtree demonstrating that Hepatitis C virus sequences from China belong to genotype 3. The tree was drawn with MEGA 4 (Build #4028), using Kimura 2-parameter as the model. The references were obtained from the Los Alamos National Laboratory HCV Databases (http://hcv.lanl.gov/content/index). Bootstrap values >70% are indicated at the nodes of the corresponding branches. Symbol ▪ indicates the sequence is from an IDU sample.
(0.77 MB JPG)
