# Supplementary figures and images for: High Prevalence and Genetic Diversity of HCV among HIV-1 Infected People from Various High-Risk Groups in China
Source: PLoS One. 2010 May 27;5(5):e10631. doi: 10.1371/journal.pone.0010631 (PMC2877711; doi:10.1371/journal.pone.0010631)

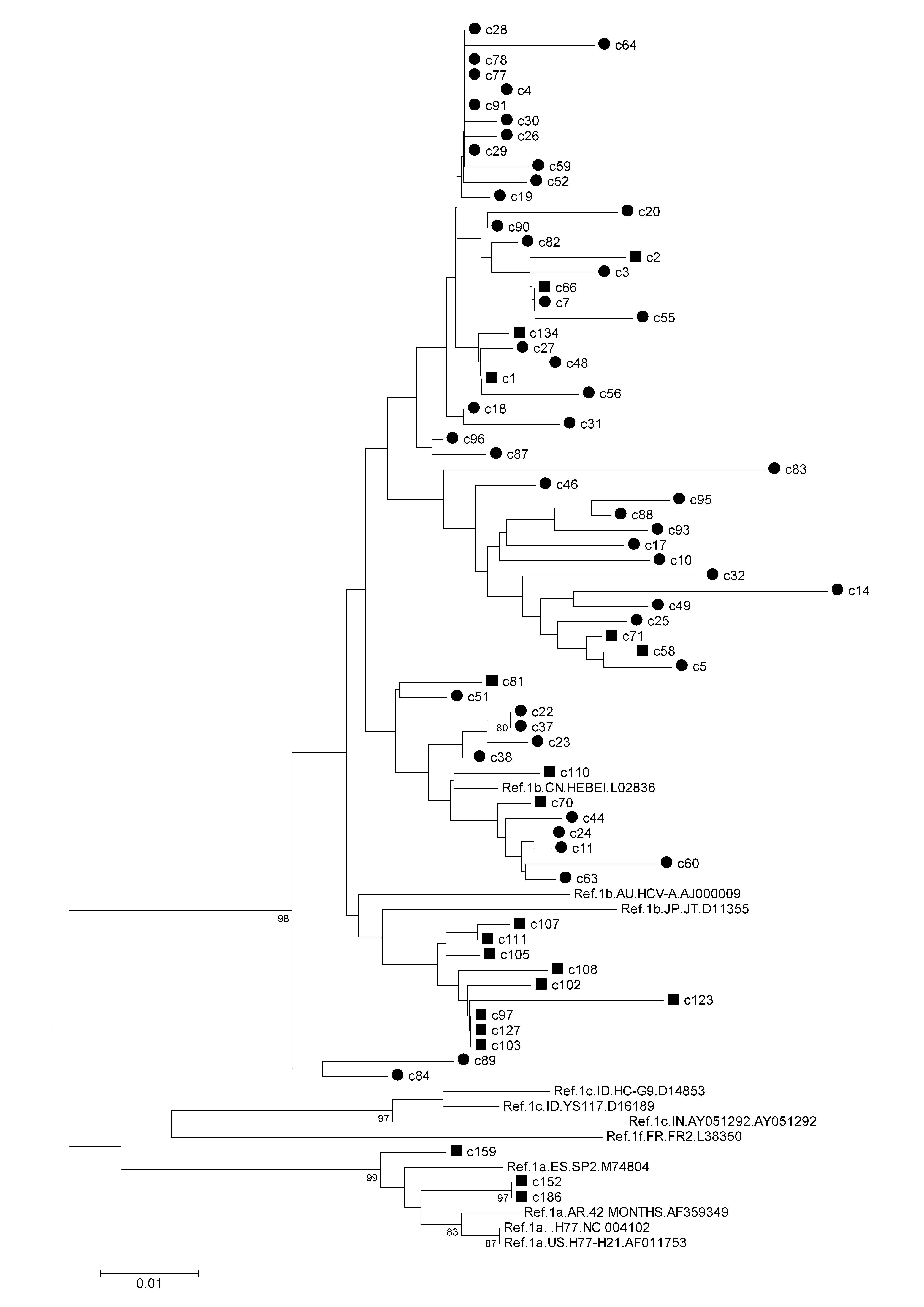

Supplement: Figure S1 — Neighbor-joining subtree demonstrating that Hepatitis C virus sequences from China belong to genotype 1. The tree was drawn with MEGA 4 (Build #4028), using Kimura 2-parameter as the model. The references were obtained from the Los Alamos National Laboratory HCV Databases (http://hcv.lanl.gov/content/index). Bootstrap values >70% are indicated at the nodes of the corresponding branches. Symbol • indicates the sequence is from a PBD sample, while ▪ is from IDU. (0.82 MB JPG) [file pone.0010631.s001.jpg]

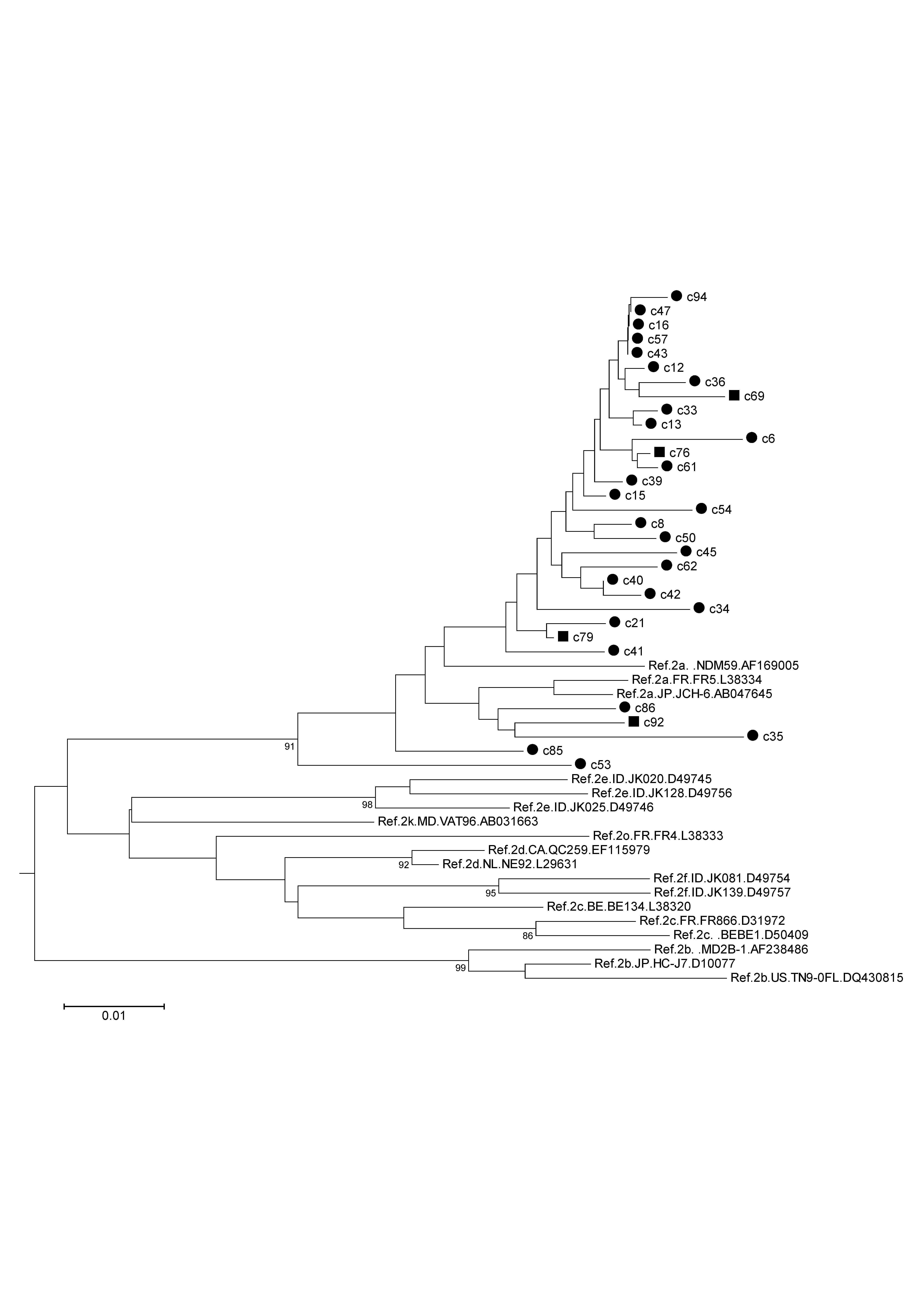

Supplement: Figure S2 — Neighbor-joining subtree demonstrating that Hepatitis C virus sequences from China belong to genotype 2. The tree was drawn with MEGA 4 (Build #4028), using Kimura 2-parameter as the model. The references were obtained from the Los Alamos National Laboratory HCV Databases (http://hcv.lanl.gov/content/index). Bootstrap values >70% are indicated at the nodes of the corresponding branches. Symbol • indicates the sequence is from a PBD sample, while ▪ is from IDU. (0.75 MB JPG) [file pone.0010631.s002.jpg]

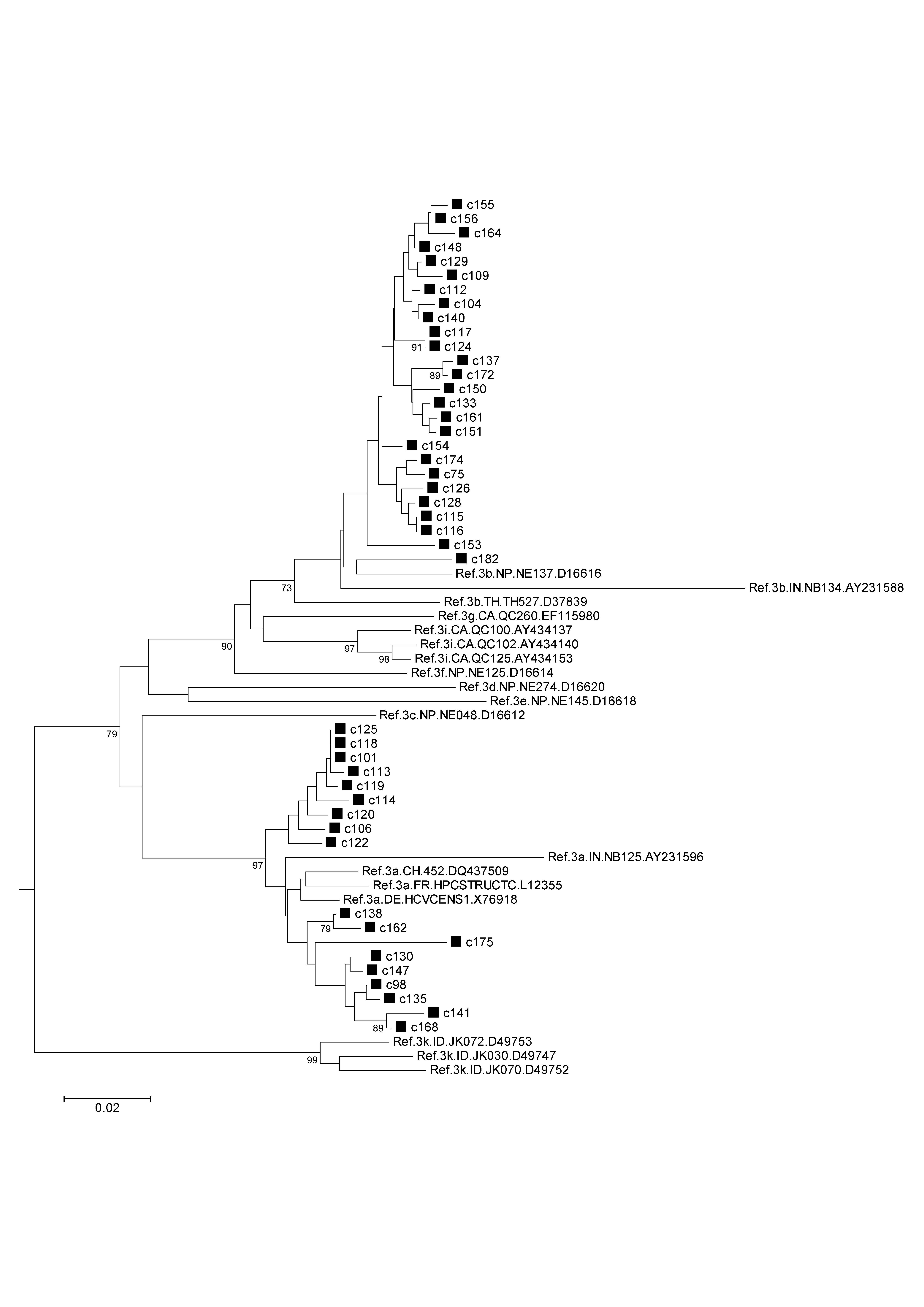

Supplement: Figure S3 — Neighbor-joining subtree demonstrating that Hepatitis C virus sequences from China belong to genotype 3. The tree was drawn with MEGA 4 (Build #4028), using Kimura 2-parameter as the model. The references were obtained from the Los Alamos National Laboratory HCV Databases (http://hcv.lanl.gov/content/index). Bootstrap values >70% are indicated at the nodes of the corresponding branches. Symbol ▪ indicates the sequence is from an IDU sample. (0.77 MB JPG) [file pone.0010631.s003.jpg]
